# Cytosolic delivery of nucleic acids: The case of ionizable lipid nanoparticles

**DOI:** 10.1002/btm2.10213

**Published:** 2021-03-20

**Authors:** Michele Schlich, Roberto Palomba, Gabriella Costabile, Shoshy Mizrahy, Martina Pannuzzo, Dan Peer, Paolo Decuzzi

**Affiliations:** ^1^ Fondazione Istituto Italiano di Tecnologia Laboratory of Nanotechnology for Precision Medicine Genoa Italy; ^2^ Department of Life and Environmental Sciences University of Cagliari Cagliari Italy; ^3^ Laboratory of Precision NanoMedicine, Shmunis School of Biomedicine and Cancer Research, George S. Wise Faculty of Life Sciences Tel Aviv University Tel Aviv Israel; ^4^ Department of Materials Sciences and Engineering, Iby and Aladar Fleischman Faculty of Engineering Tel Aviv University Tel Aviv Israel; ^5^ Center for Nanoscience and Nanotechnology Tel Aviv University Tel Aviv Israel; ^6^ Cancer Biology Research Center Tel Aviv University Tel Aviv Israel

**Keywords:** endosomal escape, intracellular delivery, ionizable lipids, LNPs, mRNA, RNA delivery, siRNA

## Abstract

Ionizable lipid nanoparticles (LNPs) are the most clinically advanced nano‐delivery system for therapeutic nucleic acids. The great effort put in the development of ionizable lipids with increased in vivo potency brought LNPs from the laboratory benches to the FDA approval of patisiran in 2018 and the ongoing clinical trials for mRNA‐based vaccines against SARS‐CoV‐2. Despite these success stories, several challenges remain in RNA delivery, including what is known as “endosomal escape.” Reaching the cytosol is mandatory for unleashing the therapeutic activity of RNA molecules, as their accumulation in other intracellular compartments would simply result in efficacy loss. In LNPs, the ability of ionizable lipids to form destabilizing non‐bilayer structures at acidic pH is recognized as the key for endosomal escape and RNA cytosolic delivery. This is motivating a surge in studies aiming at designing novel ionizable lipids with improved biodegradation and safety profiles. In this work, we describe the journey of RNA‐loaded LNPs across multiple intracellular barriers, from the extracellular space to the cytosol. In silico molecular dynamics modeling, in vitro high‐resolution microscopy analyses, and in vivo imaging data are systematically reviewed to distill out the regulating mechanisms underlying the endosomal escape of RNA. Finally, a comparison with strategies employed by enveloped viruses to deliver their genetic material into cells is also presented. The combination of a multidisciplinary analytical toolkit for endosomal escape quantification and a nature‐inspired design could foster the development of future LNPs with improved cytosolic delivery of nucleic acids.

## 1 INTRODUCTION

### 1.1 Therapeutic nucleic acids

The notion of exploiting nucleic acids (NAs) as therapeutic molecules was conceived for the first time in 1966, in a perspective paper that evoked the possible use of viruses in genetic studies and for gene therapy.^1^ However, only in the 1990s, this notion was translated into practice by a series of findings setting the stage for its use in biomedical research and, eventually, in clinical settings. First, in 1990, Wolff et al. demonstrated that the direct intramuscular injection of an in vitro transcribed (IVT) messenger RNA (mRNA) could lead to the expression of the encoded protein.^2^ Then, in 1993, the first miRNA was identified in *Caenorhabditis elegans* and, shortly after, the first mammalian miRNA—let‐7— was discovered.^3^, ^4^ Finally, in 1998, Fire and Mello discovered a fundamental mechanism in gene regulation based on RNA interference (RNAi), that was eventually acknowledged with the Nobel Prize in Physiology or Medicine in 2006.^5^ Twenty years later, in 2018, the US Food and Drug Administration (FDA) approved the clinical use of patisiran (Onpattro, Alnylam Pharmaceuticals), the first‐ever small interfering RNA drug approved for the treatment of a rare genetic disease.^6^ In about 30 years, NAs were promoted from the rank of sophisticated laboratory constructs to effective therapeutic compounds with a potentially broad spectrum of medical applications.^6^ Nowadays, with the ongoing COVID‐19 pandemic, great expectations are placed on mRNA‐based vaccines for the immunization against the SARS‐CoV‐2 virus.^7^, ^8^, ^9^

**FIGURE 1:**
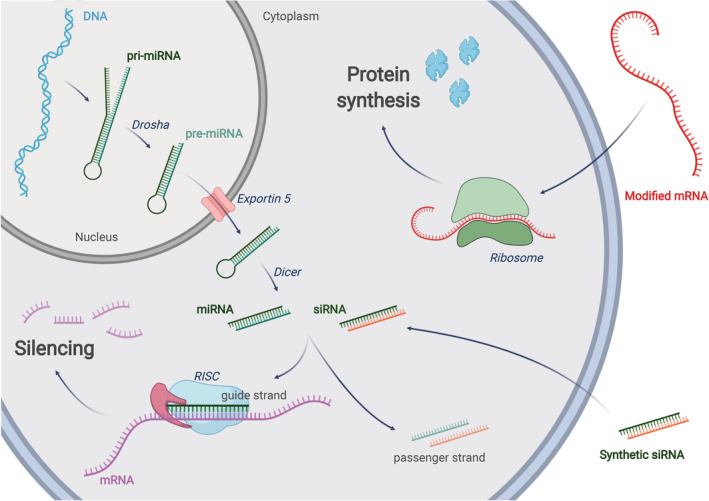
**RNA interference:** a miRNA gene is transcribed into primary miRNA (pri‐miRNA) that is further processed by Drosha to form pre‐miRNA. Exportin‐5 translocates the pre‐miRNA into the cytoplasm were it is processed by Dicer into mature miRNA. siRNAs can be obtained directly by chemical synthesis and ‐with the help of a carrier or chemical modifications‐ can reach the cytoplasm through endocytosis. In the cytosol, the guide (antisense) strand of mature miRNA or siRNA will be assembled into the RNA‐induced silencing complex (RISC). The passenger (sense) strand will be discarded. The mature RISC will find the target mRNA sequences through complementary base pairing with the guide strand. As few as 7 complementary bases (seed region) are sufficient for miRNA‐mediated RNAi, while full complementarity is usually required for siRNA‐induced silencing. Depending on the triggering molecule (siRNA or miRNA), the translation of the target gene could be repressed due to mRNA degradation or translocation to the P bodies. **mRNA therapy:** once introduced in the cytosol through an appropriate delivery method, a modified, exogenous mRNA could hijack the cell's ribosomes to be translated into a functional protein

The extraordinary medical potential of NAs resides in the fact that they can be designed to modulate the expression of any gene, including those encoding for proteins that are “undruggable” by classical small therapeutic molecules.^10^ While small molecules and monoclonal antibodies need to interact with a target protein to activate or block its function, relying exclusively on spatial structural affinity; NA‐based therapeutic agents exploit the natural cell machinery to promote gene silencing (RNAi) or protein production (mRNA).^10^ The ability of NAs to specifically knockdown or induce gene expression makes them the sole therapeutic approach capable to cope with multi‐factorial genetic diseases, cancer mutations as well as pandemic viral infections.^11^ The RNAi pathway can be exploited in different ways. For example, a gene encoding for a short hairpin RNA (shRNA) could be employed to achieve a sustained production of silencing molecules. In this case, nuclear delivery would be required, and a competition with the endogenous RNAi processing enzymes might occur. Differently, the site of action of a synthetic short interfering RNA (siRNA) is the cytosol, where the guide strand of the siRNA is loaded into the RNA‐induced silencing complex (RISC) that then binds to mRNA molecules to modulate their expression (Figure 1).^12^ Similarly, in the case of protein expression via mRNAs, the exogenous nucleic acid has to reach the cytosol where the cellular translation machinery resides.^13^

Crossing the cell membrane and localizing into the appropriate subcellular compartment have been always recognized as major obstacles to the clinical translation of NA‐based therapies. Indeed, only small, neutral, and slightly hydrophobic molecules can passively diffuse across cell membranes, while large and negatively charged molecules, such as RNAs, can only rely on active transport mechanisms, as endocytosis.^14^ This results in the confinement of NAs in intracellular organelles, as endosomes, from which NAs should rapidly escape into the cytosol to avoid progressive and fatal degradation.^15^ Viral and nonviral vectors are used for the intracellular delivery of nucleic acids.

Viral vectors refer to the use of modified viruses in which the pathogenic part of their genome has been removed, while the nonpathogenic part, which allows them to infect the cell, is retained.^16^ These vectors are extremely attractive and have helped to substantially advance the field of gene therapy because of their natural ability of inducing high transfection. Moreover, depending on the type of virus, such vectors can produce long‐term gene expression, which is currently difficult to accomplish with non‐viral methods. The properties and engineering principles of viral vectors, and of the most clinically advanced type (recombinant adeno‐associated virus) were excellently reviewed in References 17 and 18, respectively. Despite the intriguing properties and some clinical successes, the use of viral vectors is still characterized by several limitations and challenges, such as the intrinsic risk for immunogenicity, broad tropism, limited payload packaging capacity, and difficult production.

Nonviral vectors represent a valuable alternative as they are generally less immunogenic, easier to design and synthesize, and able to deliver large payloads.^19^ Nevertheless, nonviral vectors need to match some other requirements such as the biocompatibility of their constituents. Furthermore, considering that intravenous injection is the preferred route of administration, the ideal vector should maintain long circulation times and guarantee an efficient release of NAs upon reaching the target site. A vast body of literature exists on nonviral vectors for NA delivery. These vectors have been synthesized using different compositions, surface functionalities, and properties and can be broadly classified as conjugates or supramolecular assemblies. The first category comprises all those systems in which the NA is directly linked to another molecule by a covalent bond. Depending on the desired properties of the conjugate, the NA can be bound to a targeting agent, a polymer, or a hydrophobic moiety such as a lipid.^20^, ^21^, ^22^ A notable example of NA‐conjugate is givosiran, an *N*‐acetylgalactosamine‐conjugated siRNA clinically approved for the treatment of acute hepatic porphyria. On the other hand, structures formed by noncovalent interactions between NAs and other components belong to the second category. These delivery systems are generally classified based on the materials employed, which can be polymers, lipids, inorganic chemicals or a combination thereof.^23^, ^24^, ^25^, ^26^, ^27^, ^28^, ^29^, ^30^, ^31^ The recently approved vaccines against SARS‐CoV‐2, composed of mRNA encapsulated in lipid nanoparticles, belong to this class. The specific features of the different nonviral delivery systems will not be discussed in further detail since they have been reviewed elsewhere.^19^, ^32^, ^33^ It is important to highlight that nonviral vectors do not have the natural ability of viruses to efficiently overcome cellular barriers. As such, early in their engineering process, they must be optimized to favor endocytosis and endosomal escape with consequent cytosolic release of NAs. Endosomal escape can be accomplished by exploiting different strategies build‐into the vector, including the proton‐sponge effect of cationic polymers^34^; the tendency of certain lipids to form non‐lamellar phases^35^; the decoration of the vector surface with specific molecules.^36^ In the latter case, great inspiration could come from nature, as viruses and other pathogens have evolved to efficiently transfect their genetic material into host cells.^37^ Herein, we have systematically organized the literature describing the endosomal escape triggered by ionizable lipid nanoparticles (LNPs), the most clinically advanced nonviral vector for NAs delivery.^38^ After a brief overview of LNPs development from the laboratory bench to the bedside, we will critically review the work that contributed to the description of the LNPs‐mediated endosomal escape mechanism through physico‐chemical analyses, cell‐based studies, and computational simulations. Finally, we will touch on the strategies employed by viruses to deliver their nucleic acids to the cytosol, commenting on their possible exploitation to further improve the LNP technology.

### 1.2 Ionizable lipid nanoparticles for efficient nucleic acid delivery

When the first attempts to encapsulate nucleic acids were made in 1980, liposomes were already a well‐established vector for the delivery of small molecules. Initially, liposomes composed of neutral and zwitterionic lipids were employed as carriers for NA (specifically DNA), but were characterized by very low encapsulation efficiencies. Subsequently, the strategy of pre‐condensing the NAs with polycations, such as poly‐l‐lysine and protamine, was introduced to neutralize the highly negative charge of the therapeutic cargo and pack more genetic material within the aqueous core of the liposomes.^39^ In 1987, Felgner et al. described a mixture of the cationic lipid (1,2‐di‐O‐octadecenyl‐3‐trimethylammonium propane—DOTMA) and unsaturated phosphatidylethanolamine (DOPE) showing high DNA loading and efficient gene expression in vitro, setting the first milestone in the use of cationic lipids for transfection.^40^ The process was termed lipofection and the lipid mixture, which is still commercially available as Lipofectin and its more recent derivatives, is still widely used for the in vitro delivery of NAs.

Despite the in vitro efficacy and an extensive research campaign, these permanently charged lipids and the related liposomal formulation never succeeded in reaching the clinic mostly due to their unacceptable toxicity, short circulation half‐life, and unspecific association to negatively charged cellular and extracellular components.^41^ PEGylation became instrumental in masking the cationic surface charge by introducing an hydrophilic, stealth coating around the lipid particle, thus, improving systemic biodistribution and circulation half‐life.^42^ However, excessive PEGylation turned out to be detrimental for cell access and optimal subcellular distribution.^43^ Eventually, the notion of ionizable lipids was introduced whereby the quaternary ammonium head of cationic lipids was substituted with a titratable moiety.^44^ The resulting ionizable lipids present an electrostatic charge depending on the lipid pKa and the environmental pH (Table 1). The following step was indeed that of inducing the formation of a novel class of lipid nanoparticles following the self‐assembling of ionizable lipids with NAs and other components. These particles are known as the ionizable lipid nanoparticles (LNPs) and are currently recognized as one of the most advanced nonviral vectors for the efficient delivery of nucleic acids.

**TABLE 1:** The evolution of ionizable lipids from permanently charged DOTMA to FDA‐approved DLin‐MC3‐DMA

Structure	Name	pKa	Ref
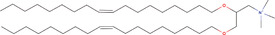	DOTMA	—	^40^
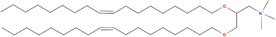	DODAP	5.8	^45^
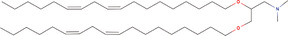	DLin‐DMA	6.8	^46^
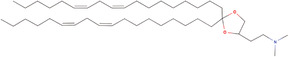	DLin‐KC2‐DMA	6.7	^48^
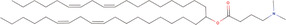	DLin‐MC3‐DMA	6.4	^49^
			

*Note*: A complete overview on the more recent ionizable lipids synthesized and used in preclinical and clinical studies is presented in Reference [50].

Ionizable lipids with an appropriate pKa would change their electrostatic charge to ensure proper vector formation, optimal in vivo circulation, and efficient cytosolic release of the therapeutic cargo. Specifically, ionizable lipids should be designed to be positively charged at acidic pH during the production of LNPs so that the electrostatic interactions with the NAs could be maximized (high NA condensation = high loading efficiency). Then, the ionizable lipids should turn into almost neutral under physiological conditions (pH 7.4) to prevent rapid sequestration by immune cells during systemic circulation (positive nanoparticles are rapidly sequestered by Kupffer cells in the liver and splenic macrophages). Also, the ionizable lipids should become positive again upon exposure to the typical acidic environment of the endosomes, thus, destabilizing the endosomal membrane and promoting the cytosolic delivery of the genetic cargo. Finally, in the cytosol, the neutral pH would favor the release of the NA from the ionizable lipids, thus, enabling their free interaction with the cell machinery.

To support the progression of LNPs from the first pre‐formulation studies in 2001, to the clinical approval of the first siRNA therapy in 2018,^6^ a multidisciplinary, translational research program was put in place by Cullis and his group based on this simple biophysical property of lipids (Table 1). Taking a closer look to the LNP development, the first ionizable lipid was 1,2‐dioleoyl‐3‐dimethylammonium propane (DODAP), whose rapid mixing with other lipids and oligonucleotides in the presence of ethanol allowed encapsulation efficiencies as high as 70%.^45^ Then, after realizing that polyunsaturated lipids could lead to more efficient transfections,^46^ LNPs were synthesized using 1,2‐dilinoleyl‐*N*,*N*‐dimethyl‐3‐aminopropane (DLinDMA). This was the year 2006 and the resulting LNPs were used to systemically deliver siRNA to nonhuman primates.^47^ Despite the successful and prolonged protein knockdown, the potency and tolerability of the system were not sufficient to proceed to advanced clinical stages. A thorough optimization of the ionizable lipids was launched with the synthesis of libraries of novel lipids, formulation and in vivo testing of LNPs aiming at knocking down hepatocyte‐derived and blood‐circulating factor VII (FVII).^48^ In 2010, this work led to the synthesis of 2,2‐dilinoleyl‐4‐dimethylaminoethyl‐[1,3]‐dioxolane (DLin‐KC2‐DMA), a potent ionizable lipid, followed shortly after by an even more efficient derivative, the dilinoleylmethyl‐4‐dimethylaminobutyrate (DLin‐MC3‐DMA).^49^

Compared to the first generation of LNPs formulated with DLin‐DMA, the DLin‐MC3‐DMA‐based LNPs showed two orders of magnitude increase in potency, defined as the dose required to achieve a 50% knockdown of circulating FVII. Such improvement, together with a better safety profile and a convenient tropism for the liver, allowed the novel DLin‐MC3‐DMA‐based LNPs to enter a Phase I clinical trial in 2012. These LNPs were loaded with an anti‐transthyretin siRNA for treating polyneuropathies induced by hereditary transthyretin amyloidosis. The positive outcome of this first human trial set the basis for further clinical development that eventually culminated in the regulatory approval of Onpattro by FDA and EMA, in 2018.^6^ Nowadays, the research on new ionizable lipids is thriving, in search for molecules with better tolerability, defined organ tropism and improved endosomal escape.^50^

It is important to highlight that since the beginning, the LNP development was supported by a coordinated research effort at the interface between academia, industry, and the clinic. This effort is still ongoing with the objective of using LNPs to target other organs than the liver and deliver larger nucleic acids, such as mRNA.^51^, ^52^, ^53^ Also, in parallel to the above described translational research effort, several groups focused on studying the fine biophysical interaction of LNPs with different cells. A great deal of work was dedicated to characterizing the endosomal escape and cytosolic delivery of the genetic materials. Quite a few authors concluded that the efficacy of LNPs in promoting the endosomal escape of nucleic acids was extremely limited with less than 2–3% of the intracellular siRNA being visualized in the cytosol.^54^, ^55^ If this modest percentage can successfully induce gene silencing in the liver, where most of the injected LNPs accumulate, it is questionable whether this approach could work for targeting other organs and diseases.^56^ Therefore, increasing the percentage of RNA escaping into the cytosol is recognized as a necessary condition to unleash the full potential of LNPs.

In the following sections, key findings on the biophysical mechanisms regulating the interaction of LNP with the endosomal machinery and the release of the genetic cargo into the cytosol are reviewed based on physico‐chemical interpretations, cell‐based studies, and computational modeling.

## 2 HOW DO IONIZABLE LIPID NANOPARTICLES OVERCOME THE ENDOSOMAL MEMBRANE?

The efficiency of an RNA therapy is influenced by several factors: the specificity of NAs; the ability of the vector to protect the NAs from biodegradation; the tropism of the vector for the diseased tissue; and the ability of the vector to release its cargo into the proper subcellular compartment. Assuming that the NA has been properly selected and designed, the efficiency of an RNA therapy depends essentially on the number of NAs reaching the intracellular target. In other words, the higher is the amount of siRNA that can be loaded on RISC, the amount of antimiR pairing with the target miRNA or the amount of mRNA engaging with the ribosome and the higher will be the therapeutic efficiency (Figure 1). All these molecular targets—RISC, miRNA, and ribosome—share the same subcellular location: the cytosol. In this scenario, the endosomal compartment represents a formidable barrier for the cytosolic accumulation of NAs, and it is recognized as the main limiting factor to their efficacy.^57^ Understanding the biophysical mechanisms regulating the cytosolic delivery of nucleic acids is fundamental to expand the realm of applications of the RNA therapies.

**FIGURE 2:**
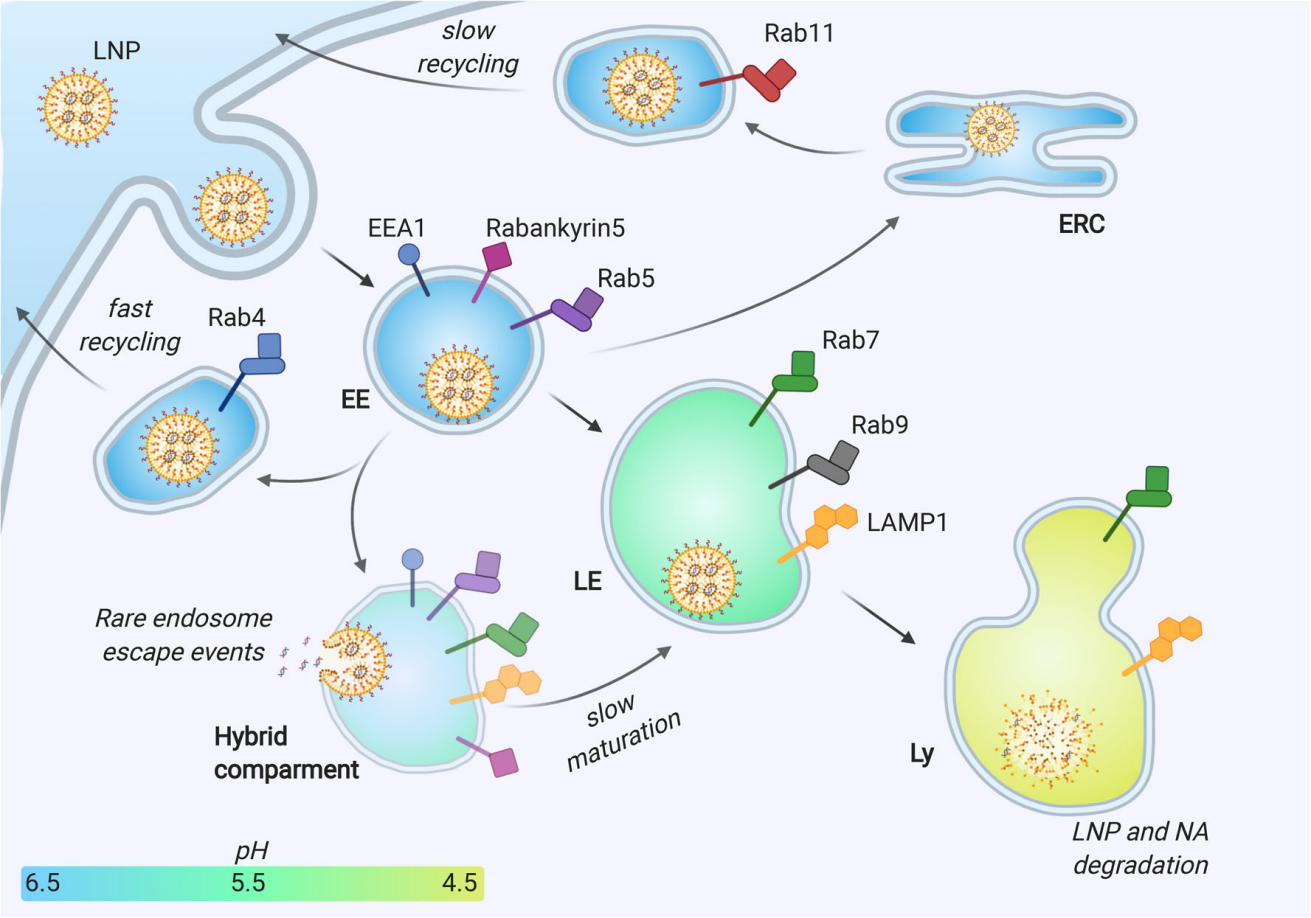
Schematic representation of the endocytic pathway, showing the different possible fates of an internalized LNP. The endocytosed LNP engulfed in an early endosome (EE) can be sent back towards the cell membrane and excreted either directly (fast recycling) or through other intracellular organelles such as the endocytic recycling compartment (ERC) (slow recycling). Alternatively, the EE matures to late endosome (LE), gradually modifying its receptors and enzymatic pool and decreasing its pH. The endosomal escape events were suggested to occur at an intermediate, hybrid compartment stage between EE and LE (see also the section Cell‐based Studies). Eventually, the LE fuses with the lysosome (Ly), whose enzymes can dismantle and degrade the entrapped LNPs and their NA payload. On the surface of endo‐lysosomal vesicles, the figure shows the main stage‐defining markers employed in the works analyzed in this review: EEA1, early endosome antigen 1; RabX, Ras‐related protein RabX (X=4, 5, 7, 9, 11); LAMP1, Lysosome‐associated membrane glycoprotein 1

**FIGURE 3:**
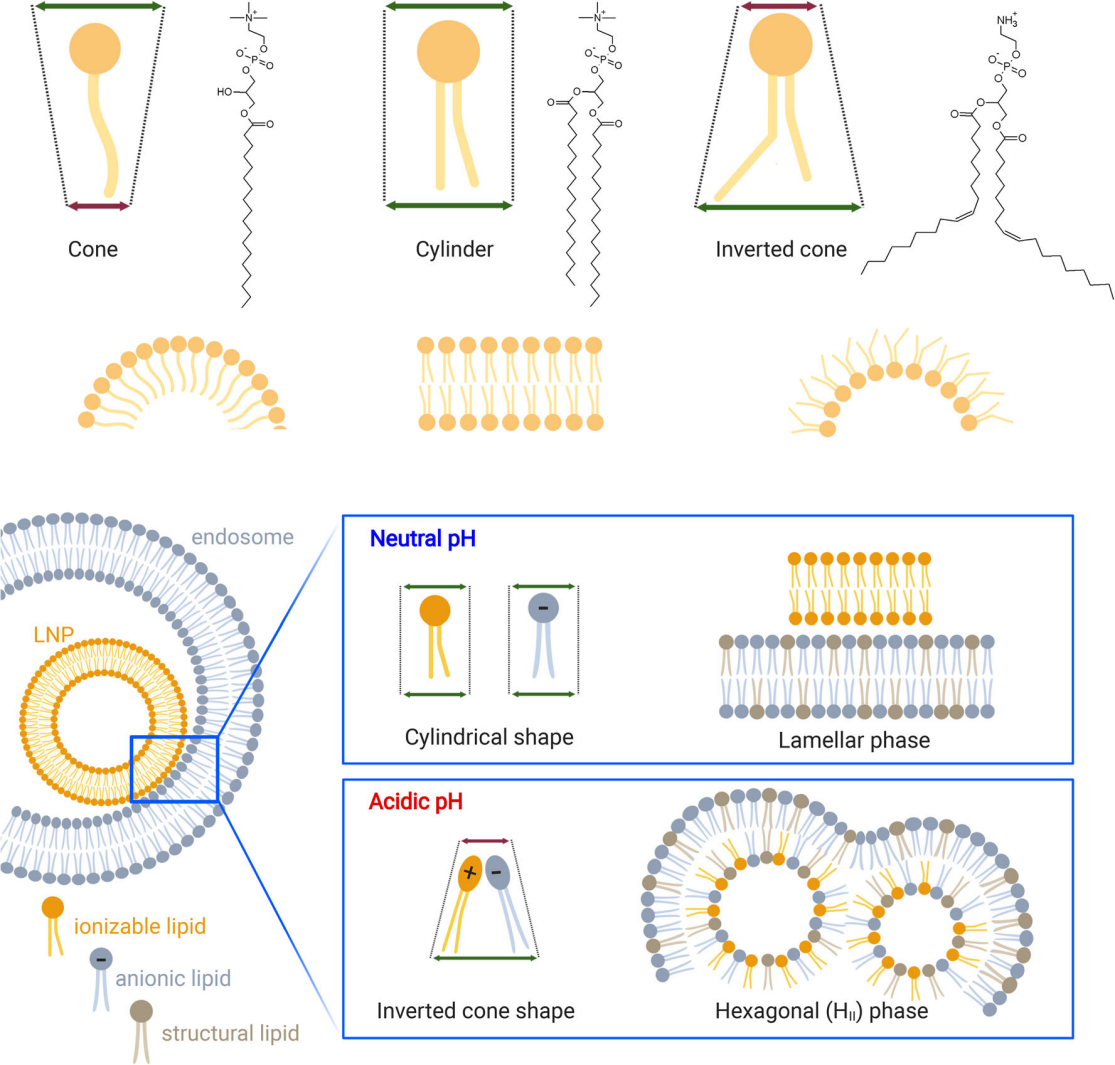
**Top:** the molecular structure hypothesis: the geometry of the lipid molecule dictates the structure of its aggregates. Cone lipids (e.g. 1‐Stearoyl‐sn‐glycero‐3‐phosphocholine) form micelles, cylindrical lipids (e.g. 1,2‐distearoyl‐sn‐glycero‐3‐phosphocholine, DSPC) form bilayers and inverted‐cone lipids (e.g. 1,2‐Dioleoyl‐sn‐glycero‐3‐phosphoethanolamine, DOPE) form hexagonal phases (H_II_). **Bottom:** the geometry of lipids might change upon mixing and ion pair formation, with consequences on the macrostructure. Protonated ionizable lipids interact with anionic lipids adopting an inverted cone shape, which promotes the formation HII phase. Non‐bilayer phases are associated with membrane fusion

The cellular uptake of LNP mainly relies on the endocytic pathway. More in detail, it has been shown that specific serum proteins adsorbed on the surface of LNPs upon intravenous injection can drive the cell internalization.^58^ This mechanism has been carefully elucidated for liver‐targeting LNPs, which are taken up by hepatocytes following the interaction between apolipoprotein E ‐adsorbed on the particles‐ and low‐density lipoprotein receptors on the cell membrane.^59^ Other receptors may be involved in the cell uptake of LNPs if a targeting ligand (e.g., an antibody) is used to decorate their surface.^60^ In general, all these cell uptake processes require, at first, the formation of early endosomes (EE), which are cellular vesicles engulfing the nanoparticles with a pH ranging between 5.5 and 6.5. These vesicles undergo a maturation process leading to a progressive reduction of the pH to 5.0–5.5, which identifies the late endosomes (LE). Eventually, the fusion with lysosomes (Ly) takes the vesicle environmental pH down to 4.5–5.5. The lysosomes are furnished with a series of enzymes such as lipases, nucleases, glycosidase, proteases, phosphatases, sulfatases that together are able to easily dismantle both the LNP structure as well as degrade the NAs^61^ (Figure 2). As such, for an effective nucleic acid delivery, a large portion of functional molecules should escape the endosomal compartment before this degradation cascade begins. Ionizable lipids, which are capable of modulating their charge depending on the environmental pH, are recognized as a key component of LNPs for the endosomal escape. As endosomal maturation starts, the heads of the ionizable lipids turn positive and start binding the negative lipids exposed on the endosomal membrane. This binding perturbs the original endosomal membrane organization leading to the formation of a nonbilayer, hexagonal (H_II_) structures inducing membranes fusion and endosomal disruption with a consequent escape of the entrapped nucleic acid (Figure 3). This proposed mechanism assumed a crucial relevance in the extensive work of optimization of ionizable lipids carried out in the last decades, and will be discussed in details here below.^62^

### 2.1 Physico‐chemical interpretation

Since 1978, Cullis and Hope pioneered the study of the physico‐chemical properties of the system linking the formation of nonbilayer structures with the “fusogenic” property of lipids.^63^ In general, lipids can self‐assemble in aqueous media to form characteristic mesoscopic phases, whose morphology is dictated by the geometry of the lipid molecule (size of the polar head, length, and unsaturation of the alkyl tails) and can depend on the temperature and presence of ions (Figure 3).^64^ Unsaturated phosphatidylethanolamine, like DOPE, are known for their inherent ability to adopt an hexagonal phase, while phosphatidylcholines are preferentially associated in bilayers.^65^ This significantly affects the transfection efficiency of these lipids in that while mixtures of DOPE and cationic lipids are potent transfection reagents (e.g., Lipofectamine and Lipofectin); the simple substitution of DOPE with a phosphatidyl choline (DOPC) completely abolish nucleic acid delivery.^66^, ^67^

When freely dispersed in water, oppositely charged lipids adopt a lamellar structure but, upon mixing, tend to form nonbilayer phases, following the reduction of the combined polar head size due to electrostatic attraction and enlargement of the hydrophobic section (Figure 3).^68^ The formation of an H_II_ structure upon contact of lipoplexes and unilamellar anionic liposomes in aqueous media was observed by ^31^P NMR, and it was accompanied by the immediate release of DNA from lipoplexes, providing a strong evidence of a link between these two events.^65^ In the same work, the authors foresee the possibility to correlate transfection efficiency with the ability of a given cationic lipid to adopt an H_II_ structure when in contact with anionic lipids. The capacity to transition from one state to another can be conveniently measured via the bilayer‐to‐H_II_ transition temperature (*T*_BH_). *T*_BH_ is defined as the temperature at which cationic (ionizable or permanent) lipid assemblies shift from the lamellar to the hexagonal phase, upon equimolar mixing with anionic vesicles. The lower is the *T*_BH_, the stronger is the tendency to adopt nonlamellar phases. The positive charge of permanently cationic lipids does not depend on pH and therefore it is always available for ion pair formation with anionic lipids. Conversely, in the case of ionizable lipids, the hydrophilic head must be in its protonated form to trigger the same process. This pH‐dependence provides a unique benefit, as an ionizable lipid with optimal pKa (around 6.5) is neutral in the circulation, preserving a bilayer structure but becomes protonated at endo‐lysosomal pH assuming an hexagonal phase upon contact with anionic membrane lipids.^49^ Thus, *T*_BH_ and pKa have been used as guiding parameters for the rational design of novel ionizable lipids.^48^ However, as pointed out by the authors, these measures do not fully account for the biological activity of ionizable lipids, whose ability to deliver NAs into the cytosolic compartment also depends on other structural features as well as the biological properties of the target cells and tissue. For instance, a flexible linker between the polar head and the lipid tails is thought to be essential to allow sufficient proximity of the cationic head and endosomal membrane lipids.^48^

### 2.2 Cell‐based studies

Cell‐based assays can be used to understand the LNP ability to induce the endosomal escape of their cargos. Working with artificial membranes in aqueous media is not sufficient to capture the complex mechanisms, thus, analytical tools supporting the visualization of endosomal escape in vitro and in vivo were developed. In this regard, Gilleron et al. described a combination of technologies to provide quantitative measures of siRNA endosomal escape following LNP transfection.^54^ The cytosolic release of siRNA was monitored by using fluorescent or gold‐labeled siRNA molecules for visualization by confocal and electron microscopy, respectively. The LNPs employed in this study included the ionizable lipid DLin‐MC3‐DMA (MC3) and showed to delay the endosomal maturation inducing the formation of a hybrid endocytic compartment, containing both early and late endocytic markers (LAMP1 + EEA1 or Rabankyrin‐5). Results revealed that only a small fraction of siRNA (1–2%) is typically released from the endosome and that the release occurs during a defined stage of endosomal progression, corresponding to the so‐called “hybrid compartment” stage (Figure 2).

Shortly after, Wittrup et al. used lipoplexes to set up an imaging approach capable to capture Alexa Fluor‐647‐tagged siRNA release from the endosomal compartment.^55^ The system was based on the acquisition of the same images with different exposure times, thus, allowing for the correct visualization of the many siRNAs concentrated inside brightly fluorescent endosomes (short exposure) and a few siRNAs escaping in the cytosol (long exposure). It was found that only a fraction of siRNA escaped from maturing endosomes (expressing RAB5, RAB7, RAB9 and not expressing EEA1 and LAMP1) shortly after the uptake of lipoplexes (5–15 min). This happened through a limited number of isolated escape events, which were coupled to a calcium spike originated from endosomal damage. It was observed that damaged endosomes rapidly recruit intracellular galectins 8 (Gal8) and 9, which are otherwise distributed in the whole cytosol under basal conditions. Exploiting the recruitment of galectins as an indicator of siRNA‐releasing endosomes, the amount of siRNA released in the cytosol was estimated upon delivery through LNPs. Indeed, the direct siRNA visualization method optimized for lipoplexes was not applicable to LNPs (formulated with a biodegradable derivative of MC3). This was probably due to the lower amount of siRNA loaded on LNPs compared with larger lipoplexes. In agreement with the previous study, the authors estimated that only 3.5% of LNP‐administered siRNA was released in the cytosol. The escape took place in a narrow time window, and at a slightly earlier stage compared to lipoplex‐delivered siRNA: a lower RAB7 expression of and no RAB9 were found on the endosomal membrane. Noteworthy, the recruitment of Gal8 was recently validated as a quantitative method for the detection of endosomal disruption triggered by cationic polymers,^69^ and sensors based on Gal8‐split luciferase fusion proteins were developed for high throughput screening purposes.^70^ Although not specifically tested on ionizable LNPs, these sensors might provide a formidable tool for the screening of new cytosol‐targeting nanoparticles.

Overall, in addition to the scarce amount of cytosolic siRNA determined, these studies agree on the narrow time frame in which endosomal escape occurs, and they both exclude that cytosolic release could occur from LE or lysosomes. Despite the different molecular signatures found on the releasing endosomes by the two groups, they both indicate a hybrid, maturing endosome as the optimal condition for siRNA escape. The pH of this compartment, although not being experimentally measured, is described as supportive of the ion‐pair mechanism observed in the physico‐chemical studies discussed in the previous section.

More recently, the use of LNPs as nucleic acid vectors was extended to mRNA.^71^ Compared to siRNAs, mRNAs have larger hydrodynamic volume and greater molecular weight. These parameters could be responsible for differences in the process of endosomal escape, which was thus investigated in the works discussed below. Sayers et al. profiled the endocytic compartments of different cancer cell lines, characterizing the luminal pH, the expression of typical markers, the morphology, and location of endosomes.^72^ Then, MC3‐LNPs were used to deliver mRNA to the studied cell lines. The differences observed in the endocytic machinery of the cell lines were correlated to a different mRNA activity. For instance, rapid endosomal maturation and lower vesicular pH positively influenced mRNA translation. In another study, only 1% of the MC3‐LNPs‐delivered mRNA was recovered in the cytosol of epithelial cells.^73^ Interestingly, it was shown that a part of the mRNA stuck in the endosomal compartments can be packaged in extracellular vesicles (EV) together with other LNP components, and exocytosed. The EVs harvested from the culture medium can induce protein expression in other cells in vitro, and in vivo upon intravenous administration. This observation leaves an open question about the relative contribution of LNPs and LNP‐shuttling EV to the systemic effects of nucleic acid therapies.

These works show that, despite being included in the composition of an approved medicinal product, the lipid MC3 is far from being the ideal candidate for cytosolic delivery of RNA drugs. Thus, the development of novel ionizable lipids is fundamental to improve this and other drawbacks of MC3, such as its nonbiodegradability (which might become an issue in frequent dosing regimens). For instance, Moderna Therapeutics developed a biodegradable ionizable lipid (lipid 5) that showed faster clearance and drastically reduced liver accumulation compared to MC3.^74^ Of interest for this review, lipid 5‐LNP allowed a 6‐fold increase in the amount of cytosolic mRNA compared to MC3‐LNPs, leading to an unprecedented 15% of internalized mRNA available for translation. The authors conclude that this improvement is not related to a higher uptake, as MC3 showed better performances in this regard, but rather to the higher fusogenic character of lipid 5, which turned in a more efficient escape and lower co‐localization with early and late endocytic markers. Similar improvements in cytosolic delivery have been obtained with other proprietary lipids, specifically developed by the company for intramuscular delivery of mRNA vaccines.^75^ These reports are only a glimpse of the ionizable lipid development activity carried out at Moderna, proven by the number of patents issued in the last years^76^, ^77^ and culminating in the use of the proprietary ionizable lipid SM‐102 for the composition of a clinical‐stage SARS‐nCoV2 vaccine.^78^ An investigation on the endosome escape mechanisms triggered by Moderna's ionizable lipids was also carried out by Patel et al.^79^ In this work, haploid cells were genetically modified to be devoid of early or late endosome maturation effectors, to investigate the role of each compartment on the translation of mRNA delivered by LNPs. Interestingly, cells lacking the late endosomal regulator Rab7a showed a dramatically reduced expression of the mRNA‐encoded protein, suggesting that the formation of late endosomal/lysosomal compartments is required for mRNA translation. Of note, the readout monitored in this study was the protein synthesis and not the visualization of cytosolic mRNA, whose escape conditions were not determined. Indeed, the formation of LE/Ly was shown to have a direct effect on translation through the activation of a signaling cascade involving mTORC1.

In addition to the development of novel ionizable lipids, several modifications to the LNPs composition have been tested in the attempt of improving cytosolic delivery. In a recent work, natural and semi‐synthetic phytosterols were employed to substitute cholesterol in the LNP composition.^80^ Through a detailed structure–activity relationship study, the authors identified sitosterol‐LNPs (eLNPs) as able to increase mRNA delivery as compared to conventional LNPs. Interestingly, the larger alkyl tail of sitosterol promoted a faceted morphology of eLNPs rather than a continuous curvature. Despite not showing a significant increase in endosomal escape compared to LNPs at early time points (4 h), eLNPs were found to have higher intracellular mobility by real‐time three‐dimensional (3D) single‐particle tracking, which might translate into a cumulative larger amount of cytosolic mRNA over time. In addition to the lipid packing defects which might enhance membrane fusion and instability, the substitution with sitosterol might evade the cholesterol trafficking machinery, previously indicated as responsible for recycling and exocytosis of a substantial portion of LNPs.^81^

Other strategies for the enhancement of endosomal escape could be borrowed from other types of nanoparticles. For instance, potent siRNA transfection and antitumor activity have been shown by core‐shell calcium phosphate (CaP)/cationic lipid nanoparticles.^82^ Once inside endosomal compartment, the low pH allows for the dissociation of CaP and the nucleic acids. The released calcium ions promote endosome disruption by raising the osmotic pressure and triggering fusion events by their binding to anionic lipids.^83^ The combination of CaP with ionizable lipids—not reported to the best of our knowledge—might provide a novel platform characterized by reduced toxicity and a dual‐mechanism of endosome escape. Another approach that enhanced the amount of cytosolic RNA consists in the sequential treatment of nanogel‐transfected cells with cationic amphiphilic drugs (CADs).^84^ In a recent report, the authors investigated if the adjuvant effect of CADs could be extended to nanocarriers having different structures and compositions.^85^ Interestingly, the cytosolic delivery mediated by MC3‐LNPs could not be improved by CAD treatment, probably due to the stable interaction between the ionizable lipid and the nucleic acid.

### 2.3 Computational modeling

The complexity of LNP's transfection dynamics and intracellular trafficking cannot be fully appreciated and elucidated by experiments only, although a broad variety of experimental techniques have been employed.^86^ Computational methods, such as molecular dynamics (MD), have often been proposed as a complementary approach. Simulations of molecular processes can be performed using physical models with different spatial and temporal resolutions, ranging from detailed, but time‐intensive, all‐atom (AA) representations to more computationally efficient coarse‐grained (CG), mesoscopic, and continuum‐mechanics models.

MD simulations in combination with cryo‐EM analyses, density measurements, and membrane fusion assays have helped to elucidate morphological features of LNPs.^87^ For instance, the CG Martini model has been used to simulate the self‐assembly of siRNA together with the ionizable lipid DLin‐KC2‐DMA (KC2), PEG‐lipids, DSPC, water, and cholesterol. Results from simulations suggested a correlation between the electron‐dense core of LNPs and the formation of inverted micellar structures resulting from the interaction of the ionizable lipids with siRNAs. However, the model could not recapitulate in full the experimental data and a new model was proposed in which siRNA molecules were packed at high concentration between juxtaposed lipid bilayers.^88^ At low siRNA concentrations, under neutral conditions, part of the ionizable lipids were proposed to segregate and form an oil droplet in the interior of the LNP. This was indeed compatible with the experimentally detected amorphous electron‐dense core. This was further confirmed by AA simulations (CHARMM36 force field,^89^) of a bilayer, consisting of POPC, KC2, and cholesterol, in water at basic, neutral, and acidic pH.^90^ Protonated KC2 (KC2H), corresponding to experimental systems at pH of approximately 4, were stably mixing with other lipid components, whereas the neutral form corresponding to experimental systems at pH of approximately 7.4 segregated into the bilayer core. Note that if KC2 is segregated in the interior of the LNP, it would not be protonated at the acidic endosomal pH. Consequently, its interaction with the endosomal membrane would be delayed or even abolished, thus, compromising the RNA released into the cytosol. As discussed in the previous sections, successful transfection and effective cargo release are indeed believed to depend on the ability of cationic lipids to interact with anionic lipids forming the endosomal membrane.

**FIGURE 4:**
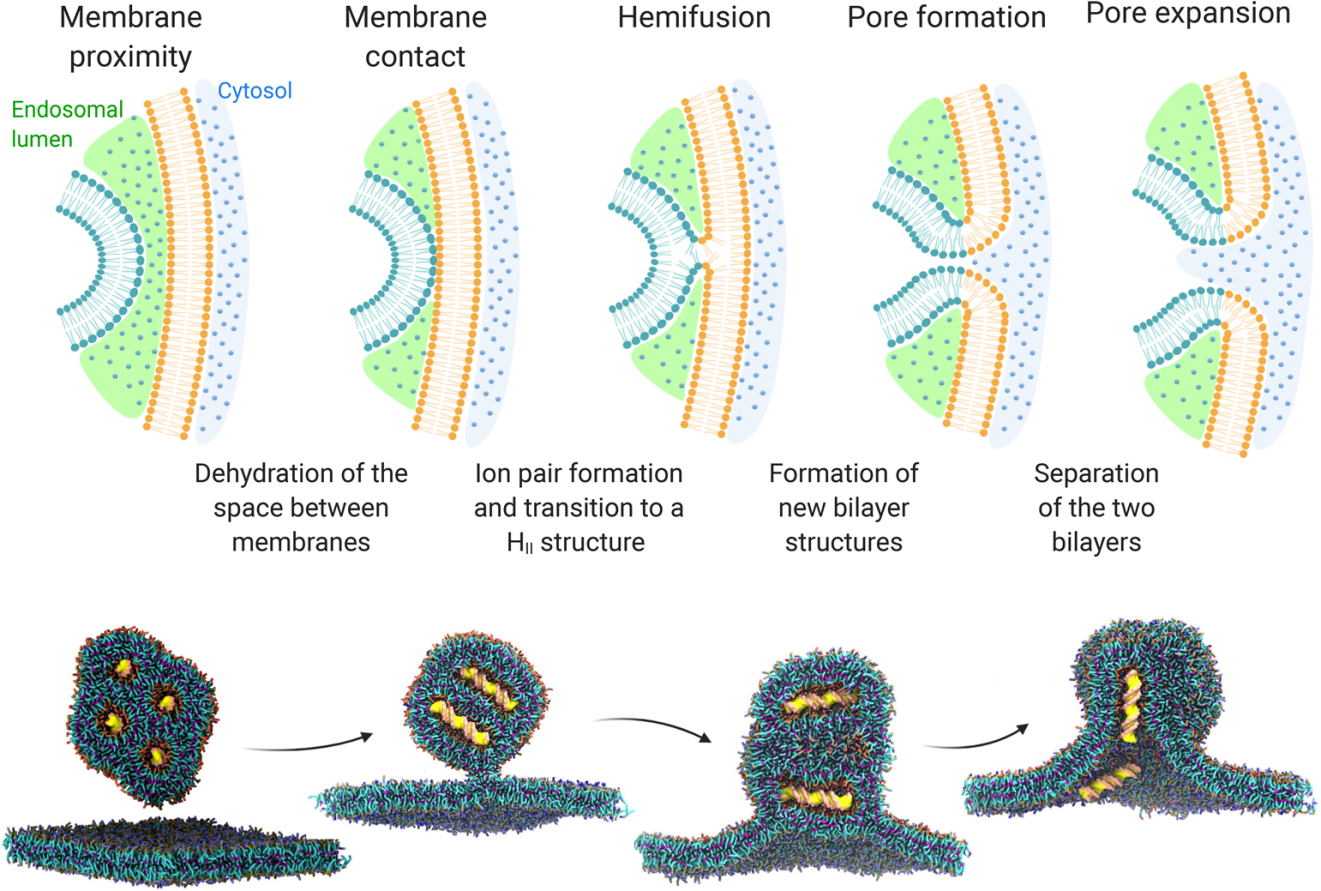
**Top:** different stages of the fusion process of two oppositely charged lipid membranes (e.g. an endosome and a LNP). **Bottom:** computational simulation of the fusion between a cationic lipoplex loaded with DNA (yellow) and a negatively charged membrane. This panel has been adapted and reproduced with permission from ^91^

Only a handful of reports describe the mechanism of LNP‐mediated endosomal escape by computational approaches. The fusion between lipid membranes, including lipid nanoparticles, proceeds through a series of energy‐demanding intermediate stages, including the close membrane contact; the formation of a hemifusion structure, where the two membranes merge into one; the formation of a fusion pore; and, eventually, the pore expansion (Figure 4).^83^ Each step represents a kinetic barrier to cross and several mechanisms can lower such barriers supporting fusion.

The fusion of DNA‐lipoplexes with the endosomal membrane has been recently investigated by CG simulations, providing insights on some of the key determinants for a successful transfection: vector size, lipoplex, and endosomal lipid composition.^91^ The Martini force‐field was used to model a lipoplex consisting of DOTAP, as the cationic lipid, a double strand of DNA, and DOPE, as the helper lipid (1:4 DOTAP/DOPE ratio), and a mimic of the endosomal membrane consisting of PC and PS lipids (4:1 ratio). The lipoplex model was validated against small‐angle X‐ray scattering (SAXS) data available in the literature. Two alternative transfection pathways were documented: the perpendicular pathway, where DNA would align parallel to the endosomal membrane, unzipped from lipids, while a pore opens concomitantly; the parallel pathway, where DNA strands oriented normally to the membrane are quickly ejected after the opening of a pore.

As previously observed experimentally,^46^ unsaturated lipid tails promoted fusion with the endosomal membrane compared to saturated lipids. The particle size also played a role, as small lipoplexes tend to be more fusogenic to alleviate the state of stress and higher potential energy associated with the larger surface curvature.

More recently, AA simulations (OPLS AA forcefield,^92^) demonstrated the higher fusogenicity of LNPs with the endosomal membrane if alkyne groups are incorporated in the lipid tails.^93^ Alkyne‐containing lipids (A6) were more inclined to protrude tails out of the LNP, diffuse laterally, sprout, and flip–flop than cKK‐E12, an ionizable lipidoid previously developed by the same group.^94^ All these events are involved in the process of membranes fusion. Data were confirmed experimentally through biophysical and cell‐based analysis, making this paper one of the few examples in the literature employing multiple techniques to investigate the cytosolic delivery through LNPs. More specifically, the combination of A6 with cKK‐E12 improved LNPs fusion with isolated endosomal vesicles and increased the amount of cytosolic mRNA detected in primary hepatocytes.

However, none of these studies exhaustively explored the contribution of electrostatic interactions in endosomal escape, related to the charged headgroups in LNPs and the asymmetric distribution of charges in the endosomal membrane.^91^, ^93^ First, MD simulations were conducted after the removal of water molecules at the interface between the nanoparticle and the endosomal membrane. This fictitious dehydration at the contact site indeed represents a kinetic barrier to membrane fusion that could not be observed using unbiased simulations.^95^ Thus, the contribution of charged lipid headgroups to the dehydrated interface condition remains unclear. Second, the endosomal membranes built for simulations had an identical lipid composition in the inner and outer leaflets, not accurately representing the complex, asymmetric biochemistry of the system.^96^

Some insights on the role of electrostatic interactions can be inferred via CG simulations conducted on dendrimers interacting with an asymmetrically charged membrane. Dendrimers, as polycationic particles, may experience electrostatic driving forces in a similar way to LNPs. Anionic lipids on the external endosomal leaflet promote the embedding of dendrimers into the membrane.^97^ Upon membrane crossing, the direct interaction with anionic lipids promotes the dissociation of siRNA from dendrimers, as determined by steered AA simulations.^98^

Dendrimers, however, also exploit peculiar electrostatic‐driven mechanisms to promote the cytosolic release of nucleic acids, such as proton sponge effect and polymer swelling. These events will increase the osmotic pressure and the endosomal membrane tension. Such critical stress will be relieved through pore formation and expansion, that is the intermediate state for the endosomal escape of genetic material.^97^ Thus, the role of electrostatic interactions in both the complexation of genetic material and endosomal escape with LNPs may differ from that of dendrimers and stays an open chapter yet to be written.

## 3 HOW DO VIRUSES OVERCOME THE ENDOSOMAL MEMBRANE?

Pathogens have learned to efficiently tackle the multiple barriers diminishing the efficient delivery of nucleic acids. Viruses are lifeless infectious particles that can only reproduce inside a host cell. Lacking their own metabolism, viruses have evolved to enter cells of host organisms (from all domains of life) to exploit their resources and reproductive machinery.^99^, ^100^ More specifically, viruses are able to evade the host immune system, reach specific cells, cross cellular barriers and, eventually, replicate. All viruses contain nucleic‐acid genomes (RNA or DNA), which are packaged with proteins encoded by the viral genome. Viruses vary in shapes and sizes, ranging from 18 to approximately 2000 nm in diameter, and can be divided into two main categories: enveloped viruses, which have a lipid membrane (envelope) derived from the host cell; and nonenveloped viruses, lacking such membrane.^101^ In this section, the strategies utilized by viruses to tackle cellular barriers in the delivery of genetic materials are discussed, and their implications in the rational design of nonviral vectors are also addressed.

Viral cell entry begins with attachment to the host cell, usually to a cell surface receptor which triggers cell uptake and delivery of the viral genome to the cytoplasm. The cell surface moiety to which viruses bind can be either a protein, a lipid or a carbohydrate.^37^, ^100^ Following binding, viruses use two main routes for cell entry: endocytic or nonendocytic.^102^, ^103^ The endocytic route includes transport in clathrin‐coated vesicles or pits, caveolin‐mediated endocytosis, macropinocytosis, and clathrin‐ and caveolae‐independent pathways.^37^, ^104^ The nonendocytic route involves direct plasma membrane crossing of the genetic cargo.^102^ Additional viral entry routes include direct cell to cell contacts known as the “virological synapse” used by retroviruses such as human T‐cell lymphoma‐leukemia virus type 1 (HTLV‐1) and HIV‐1.^102^ Viruses can also enter and exit cells without crossing membranes via transcytosis—a mechanism which utilizes vesicular transport.^102^

If the endocytic route is involved, the virus requires a well‐timed endosomal escape to avoid the recycling into the extracellular space or degradation in the harsh lysosome environment.^105^, ^106^ For this purpose, enveloped viruses (such as Filoviridae, Arenaviridae, and Orthomyxoviridae) use specific surface proteins which undergo pH‐induced conformational changes to facilitate fusion of the viral envelope with the endosomal membrane.^37^ On the other hand, nonenveloped viruses (e.g. Adenoviridae, Parvoviridae, Picornaviridae, Reoviridae) exploit membrane‐modifying proteins to disrupt the endosomal membrane (through pore formation or in a detergent‐like fashion) and release the genomic content directly into the cytoplasm of the target cells.^37^, ^107^

### 3.1 Viral fusion proteins

**FIGURE 5:**
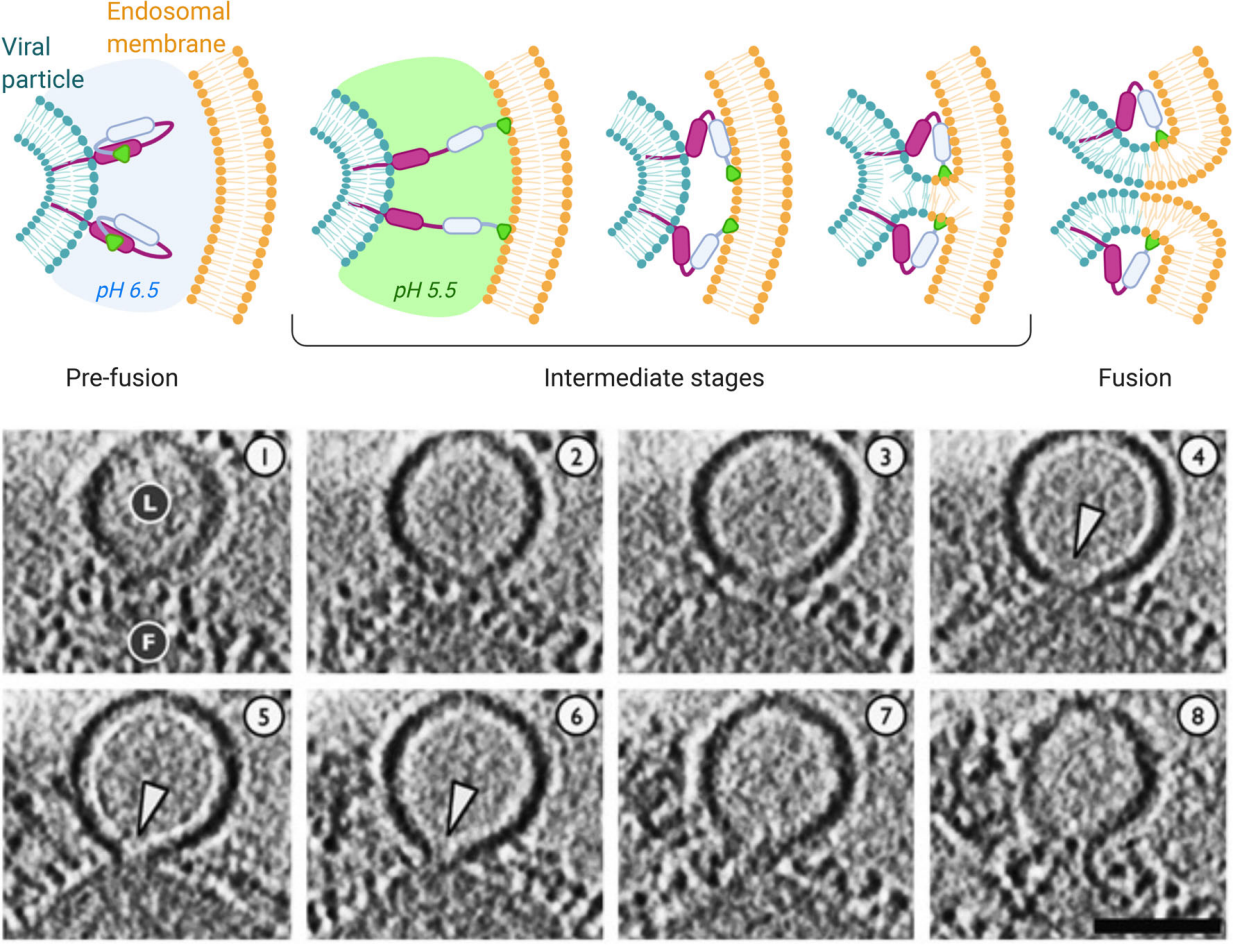
**Top:** a representation of the general mechanism of fusion between an enveloped virus and the endosomal membrane, mediated by a pH‐dependent fusion protein. In the pre‐fusion stage, the hydrophobic segment responsible for membrane binding is hidden within the protein. An external trigger (in the case of this example the pH acidification) induces a conformational change exposing the hydrophobic peptide that anchors the endosomal membrane. The protein then tends to refold to a more stable conformation, and by doing so it increases the proximity of the viral envelope and the endosomal membrane, promoting the mixing of lipids and membrane fusion. **Bottom:** cryo‐electron tomography sections (5.3 nm‐thick) of a fusion event between influenza virus (F) and a liposome used as a model membrane (L) at pH 5.5. White arrowheads indicate the formation of fusion pores. Scalebar 50 nm. This panel has been adapted and reproduced with permission from ^111^

As previously discussed, the interface between the two approaching membranes has to be dehydrated for fusion to initiate.^95^ Repulsive hydration forces do not allow membranes to come in contact without a trigger. Fusion proteins embedded in the envelopes of viruses help crossing this barrier. Although there are several types of fusion proteins (classes I, II, and III), they share some common 3D structural motifs and mechanisms of action. Ligand binding, proteolytic cleavage or low pH induce important conformational changes of the protein, which result in the exposure of the fusion peptide portion **(**Figure 5 **)**. The fusion peptide is a hydrophobic segment that can engage the host membrane, creating a bridge between the two bilayers. This leads to an increased proximity of the viral and host membrane, and eventually enables fusion following rearrangements of membrane lipids.^108^, ^109^ For example, the haemagglutinin subunit HA2 of influenza virus facilitates fusion via a highly conserved sequence of hydrophobic amino acids in its N terminal region (The C terminal end is embedded in the viral membrane). At endosomal pH, the protein refolds to expose the hydrophobic N terminal region. This triggers the fusion of the viral membrane with the endosomal membrane and subsequent viral nucleic acid release into the cytosol.^110^, ^111^ The pH required for the conformational change depends on the virus type. While some viruses such as Semliki Forest virus (SFV), fuse with the early endosomal membrane at relatively high pH (approximately pH 6), others such as the influenza virus fuse with late endosome membrane at a lower pH.^112^

### 3.2 Lessons for nanoparticle rational design

There are great differences between viruses in time and efficiency of cell entry, defined as the percentage of viruses entering the cell out of those adhering to the surface. Some viruses enter cells with great efficiency and speed (>50% efficiency in a few seconds), such as the adeno‐associated virus serotype 2, SFV, and influenza, while for others the process is slower (0.1% efficiency in several minutes), such as the HIV‐1.^102^, ^113^ Similarly, the kinetics and efficiency of endosomal escape varies with the viruses and depend on viral structural features. For instance, a lipid composition of the viral envelope affects the endosomal escape performances.^102^ The viral envelope derives from the host cell membrane. However, differences in the lipid composition of the two suggest that some viruses “select” lipids to build a more fusogenic envelope.^114^ For instance, the composition of influenza virus envelopes was found to be enriched in phosphatidyl ethanolamines (PE) and phospatidyl serines (PS) as compared to the host membrane, where phosphatidyl cholines (PC) were more represented.^115^ Both PE and PS promote fusion, as the former increases membrane fluidity while the latter improves the interaction with the annexins of the host. Therefore, the lipidomic analysis of viruses might provide useful hints for the design of LNPs with improved fusogenicity.

In addition to envelope lipids, membrane fusion proteins, or derived peptides, might be exploited to improve non‐viral nucleic acid delivery. The first viral peptide used for enhanced endosomal release was the HIV1‐TAT.^116^ Ever since, several viral‐derived or viral‐inspired synthetic analogs were reported to enhance the delivery of nucleic acids.^112^ The mechanism of action of TAT is related to its positive charge (derived from arginine and lysine residues) that leads to endosomal membrane destabilization via direct interaction with the negatively charged membrane lipids.^112^ Similar features were also shown by the human papillomavirus L2 capsid peptide, which is characterized by a positive charge and a hydrophobic domain.^117^ In other cases, viral peptides exploiting the cited pH‐driven conformational change have been employed. HA2 has been used to enhance the delivery of DNA following its incorporation to transferrin‐polylysine‐DNA complexes.^118^ The influenza HA2 fusion protein is a class I fusion protein; as such the α‐helical structure is crucial for the facilitation of pH‐dependent membrane fusion.^119^ Inspired by HA2, several synthetic pH‐sensitive α‐helical peptides were designed and their structure–activity correlation was studied.^120^ For example, GALA, a 30 amino acid peptides that switch conformation from random coil to α‐helix at pH 5, was shown to facilitate endosomal escape of nucleic acid delivered by cationic dendrimers.^121^ Newer derivatives were developed, such as KALA, which is positively charged and therefore also enables complexation of nucleic acids,^122^ and several others including INF7, EBI, and CADY.^123^, ^124^, ^125^ Exploiting the same mechanism, the glycoprotein G of the vesicular stomatitis virus was used for the enhanced gene delivery through liposomes.^126^ Finally, some viral derived peptides can mediate membrane fusion in a pH‐independent manner, such as peptides derived from the HIV‐1 gp41 protein.^127^

A more detailed understanding of these mechanisms could help to identify nature‐inspired strategies to improve the performance of LNPs. Previous studies suggest that stable contact between oppositely charged lipid heads can only occur upon dehydration of the interface between the LNP and the endosomal membrane, and membrane destabilization is required for fusion to proceed (Figure 4). As extensively discussed in this work, ionizable lipids induce the cytosolic release of nucleic acid by forming non‐bilayer structures upon ion pairing with endosomal anionic lipids. Among the viral peptides here presented, GALA and its derivatives could provide LNPs with a membrane fusion mechanism complementary to the one of ionizable lipids. LNPs may indeed benefit from the synergic action of such peptides, which help establish the first contact between the facing membranes through the formation of biological anchors. Coherently with this vision, a recent work described the development of a novel LNPs decorated with GALA‐cholesterol conjugate.^128^ The combined effect of the ionizable lipid YSK05 and GALA improved the endosome escape as compared to a control system (composed of the cationic lipid DOTMA and GALA). In another work, the virus‐derived peptide KALA was conjugated to a lipid chain and included in LNPs structure for mRNA delivery.^129^ The combination of KALA and ionizable lipids promoted the cell uptake and mRNA translation compared to controls (LNPs equipped with another peptide, or LNPs formulated without ionizable lipids). Although a mechanistic analysis of cytosolic delivery was missing, these reports support the notion of combining ionizable lipids with virus‐inspired agents to promote endosomal escape.

## 4 CONCLUSIONS AND OUTLOOKS

Different lights have been used to illuminate the mechanisms regulating the endosomal escape of therapeutic RNA loaded into ionizable lipid nanoparticles. The body of work presented in this review includes physico‐chemical and biological evidence as well as in silico modeling data and contributes to our understanding of intracellular RNA delivery. Overall, it is recognized that promoting the instability of endosomal membrane is a fundamental step. This has been realized by designing LNPs with a faceted morphology; employing lipids with a higher tendency to diffuse, protrude, and flip–flop; or biomimicking viruses using complementary escape mechanisms, such as fusion‐inducing peptides. Today, only minimal amounts of siRNA are released into the cell cytosol. However, this appears to be sufficient to achieve potent silencing and has dictated the successful advancement of LNP from the laboratory benches to the clinic. However, as our understanding of the complex mechanisms regulating the behavior of cells grows, it becomes clear that siRNA, mRNA, and cocktails of nucleic acids should be more efficiently delivered to positively address a variety of disorders, including cancer, cardiovascular, neurological, and infectious diseases. To specifically address the cytosolic delivery issue, the refinement of high‐throughput cellular screening systems for the quantitative detection of intra‐cytosol nucleic acids is urgently needed, and should be coupled with the traditional in vivo efficacy assays. The design of novel nanoparticles with higher RNA delivery efficiency would require a coordinated, multidisciplinary effort focusing not only on the design and testing of new ionizable lipids, but also on the integration into nanoparticles of additional components promoting the endosomal destabilization, possibly taking inspiration from virus‐inspired escape mechanisms.

## AUTHOR CONTRIBUTIONS

**Michele Schlich:** Conceptualization; data curation; formal analysis; investigation; methodology; visualization; writing‐original draft; writing‐review & editing. **Roberto Palomba:** Conceptualization; data curation; formal analysis; investigation; writing‐original draft. **Gabriella Costabile:** Data curation; investigation; writing‐original draft. **Shoshy Mizrahy:** Data curation; formal analysis; writing‐original draft. **Martina Pannuzzo:** Data curation; formal analysis; investigation; writing‐original draft. **Dan Peer:** Conceptualization; supervision; writing‐review & editing. **Paolo Decuzzi:** Conceptualization; funding acquisition; methodology; resources; supervision; writing‐review & editing.

### PEER REVIEW

The peer review history for this article is available at https://publons.com/publon/10.1002/btm2.10213.

## Data Availability

Data sharing is not applicable to this article as no new data were created or analyzed in this study.
